# Family Shopping Basket Intervention: A Strategy to Reduce Obesity in Prepubertal Children

**DOI:** 10.3390/jcm14010227

**Published:** 2025-01-03

**Authors:** Rocío Escartín, Beatriz de Peray, Yolanda Couto, Abel Martínez-Mejias, Raquel Corripio

**Affiliations:** 1 Pediatric Department, Consorci Sanitari de Terrassa, 08227 Terrassa, Spain; rescartin@cst.cat (R.E.); amartinez@cst.cat (A.M.-M.); 2 Pediatric Endocrine Department, Hospital Universitari Parc Taulí, 08208 Sabadell, Spain; beatrizdeperay@gmail.com; 3 Pediatric Gastroenterology, Hepatology and Nutrition Department, Hospital Universitari Parc Taulí, 08208 Sabadell, Spain; ycouto@tauli.cat; 4 Pediatric Endocrine Department, Hospital Universitari Parc Taulí, Institut d’Investigació i Innovació Parc Taulí, Universitat Autònoma de Barcelona, 08208 Sabadell, Spain

**Keywords:** childhood obesity, family-based treatment, shopping habits, grocery basket

## Abstract

**Background/Objectives:** The goal of childhood obesity treatment is to benefit the physical and mental health of children who suffer from it and to prevent complications, improving their quality of life and ensuring adequate development. Family-based interventions are demonstrating positive results, especially in prepubertal children. The aim of our study was to evaluate the effectiveness of a family grocery basket intervention for the treatment of childhood obesity in a Spanish primary care office. **Methods:** A randomized controlled trial comparing a family grocery basket intervention through the analysis of unhealthy products included in the grocery receipts that families bring to the primary care office, in comparison with the usual interventions. **Results:** Ninety-one children participated in the study (intervention group: *n* = 60, control group: *n* = 31). After one year of follow-up, a relevant weight loss with a decrease of ≥0.5 SDS in the z-IMC was obtained in 60.6% of the total sample. In the intervention group, there was a significant decrease in the number of unhealthy products in the family grocery basket and a lower percentage of hypertension and severe obesity than in the control group. In families in which there was a significant decrease in the consumption of unhealthy products, a higher percentage of weight loss was observed. **Conclusions:** Intervention in the family grocery basket through receipts is an original, simple and effective tool for family-based treatment in childhood obesity.

## 1. Introduction

Childhood obesity, given its high prevalence and the health and social problems it entails, is one of the most important public health challenges of our time [1]. In 2020, 150 million children and adolescents aged 5–19 years had obesity, and it is estimated that this number may rise to 254 million by 2030 [2]. This is a problem that particularly affects developing countries, where a concerning increase has been observed over the last years [2,3].

The objective of treating childhood obesity is to benefit the physical and mental health of children who suffer from it and to prevent complications, improving their quality of life and ensuring appropriate development [4,5]. The mainstay of childhood obesity treatment is to achieve behavioral change, leading to a long-term healthy lifestyle [5]. Family-based interventions have shown efficacy in childhood obesity, obtaining a correct weight evolution [6,7,8], improvement in blood pressure and lipid profile and decrease in insulin resistance [9].

Considering that family is a nuclear element in the pediatric population, it is necessary to continue research in the line of improving family lifestyles. It is essential to find tools that facilitate this task for primary care teams. Our study aims to evaluate the efficacy of an intervention in family shopping habits.

The main objective of this study was to analyze the effectiveness of a direct intervention in the shopping habits of families in a primary care office in Spain, advising them to avoid the purchase of unhealthy products that appear on their grocery basket receipts, in prepubertal children with obesity.

## 2. Materials and Methods

### 2.1. Study Design

This is an open, prospective, randomized and controlled clinical trial that compares the benefit of an intervention that focuses on family grocery receipts compared to the usual interventions performed as routine in a primary care pediatrics service in families of prepubertal children with obesity. The structure of the SPIRIT guideline for clinical trials [10] was followed. This study followed the CONSORT guidelines for reporting randomized controlled trials [11].

### 2.2. Participants

Prepubertal children with obesity belonging to the reference population of the primary care centers of the area of Terrassa, a city in Spain, participated. Patients who met the inclusion criteria were randomly assigned to the control or intervention group.

We focused on prepubertal children, with whom family-based treatment of obesity seems to be more effective [12].

Accepting an alpha risk of 0.05 and a beta risk of less than 0.2 in bilateral contrast, a total sample size of 116 individuals was calculated, assuming an initial proportion of 0.99 and a final proportion of 0.9, estimating a 15% loss rate.

Inclusion criteria

- Age between 6 and 10 years old.
- Body mass index (BMI) ≥ 97th percentile according to the growth curves and tables of the semi-longitudinal study by Hernández et al. [13,14].
- Informed consent signed by parents or caregivers.

Exclusion criteria

- Tanner pubertal stage higher than 1 [15,16].
- Presence of endocrinological disorders.
- Syndrome in association with obesity.
- Inflammatory disease in the ten days prior to the beginning of the evaluation.
- Intake of drugs that influence weight.
- Absence of consent from the parents or caregivers.

### 2.3. Initial Evaluation

#### 2.3.1. Anamnesis

In the initial evaluation, a detailed clinical history was taken, and the following variables were obtained:

- Weight and length at birth.
- Anthropometry of parents.
- Family history of type 2 diabetes.
- Demographic variables: sex, country of origin and socioeconomic level, based on the territorial socioeconomic index (IST) of their home location [17].
- Family and social context: separation or not of parents, single-parent family, number of cohabitants at home.

#### 2.3.2. Clinical Evaluation

Anthropometric measurements were taken in duplicate by the same observer, with the patient in underwear and without shoes. The mean of the measurements was used.

- Body weight: measured in kilograms with a scale adjusted in 0.1 kg intervals.
- Height: measured in centimeters with a stadiometer adjusted in 0.1 cm intervals.
- Abdominal perimeter, measured at the midpoint between the last rib and the upper iliac crest, with a tape measure adjusted in 0.1 cm intervals.
- BMI was calculated according to the following formula: weight (in kilograms) divided by the square of height (in meters).
- Triponderal mass index (TMI) was calculated according to the following formula: weight (in kilograms) divided by height cubed (in meters) [18].
- Blood pressure was determined in triplicate by adjusting the cuff measurement to the circumference of the right arm of each patient while lying. The value recorded was the result of the mean of the readings. Blood pressure was assessed using the reference tables of the American clinical practice guideline [19], which are those currently recommended in our setting.
- Pubertal development was assessed according to the Tanner classification (1 = prepubertal) [15,16].
- Special attention was paid to the existence of acanthosis nigricans.

#### 2.3.3. Laboratory Analysis

Blood samples were obtained from the ante ulnar vein of each patient after 12 h of fasting. After extraction, the plasma was separated from the cellular package by centrifugation at 2500 rpm for 10 min and then stored at −80 °C until analysis. The following parameters were analyzed:

- Plasma glucose: measured by the hexokinase method, with variation coefficients of 1.9–2.1%.
- Insulin: measured by electrochemiluminescence, with inter-series variation coefficients of 2.6–2.8%.
- Total cholesterol and fractions: values obtained using the cholesterol oxidase, esterase, peroxidase method.
- Triglycerides: values obtained using the molecular absorption spectrometry/colorimetric method.
- Liver enzymes AST (aspartate aminotransferase) and ALT (alanine aminotransferase) were measured using molecular absorption spectrometry with pyridoxal phosphate.
- Uric acid: values obtained using uricase/peroxidase molecular absorption spectrometry.
- Inflammatory parameter interleukin 6: determined by immunoassay.

### 2.4. Follow-Up

In both groups, after the initial evaluation, each patient was recommended (orally and in writing) a balanced normo-caloric diet adjusted to his or her age. The use of screens was limited to a maximum of 2 h per day, and a moderate exercise program of at least 30 min three times a week was negotiated.

Children in both groups were seen in the office every 3–4 months, reevaluating anthropometric and clinical parameters. In both groups, at each visit, the recommendations on food, screens and exercise established at the initial visit were repeated.

In the intervention group, at each visit, families showed the grocery receipts for the month prior to the visit. After reviewing them, the unhealthy products purchased that should be avoided in future purchases were indicated and recorded in grams and kilocalories. These were sliced bread, pastries, cookies or other sweet doughs, bacon, sausages, pâté, condensed milk, cream, sweet desserts, margarine, butter, industrial sauces, processed juices, soft drinks, milkshakes, chocolate and its derivatives, ice cream, candy, convenience foods and salty snacks.

Analytical re-evaluation was performed in both groups after one year.

Significant weight loss was considered in patients who obtained a decrease of at least 0.5 standard deviations in BMI percentile, given its relationship with the improvement of cardiovascular health [20,21].

### 2.5. Statistical Methods

Data were expressed as means ± standard deviations (SDS) for quantitative variables and as percentages for categorical variables unless otherwise indicated. A regression model was performed with BMI SDS as the dependent variable and age, sex, country of origin, number of cohabitants, family situation, socioeconomic level, family history of diabetes, parental anthropometry, baseline characteristics and consumption of unhealthy products as independent variables. The percentage change of the parameters after one year was calculated as a rate, defined as follows: (final value after one year—initial value)/initial value × 100). To compare variables before and after the intervention, the t-test or Wilcoxon test was used. Pearson or Spearman correlation coefficients were calculated. The level of statistical significance was set at 0.05. Analyses were performed using SAS v9.4, SAS Institute Inc., Cary, NC, USA.

## 3. Results

**Figure 1:**
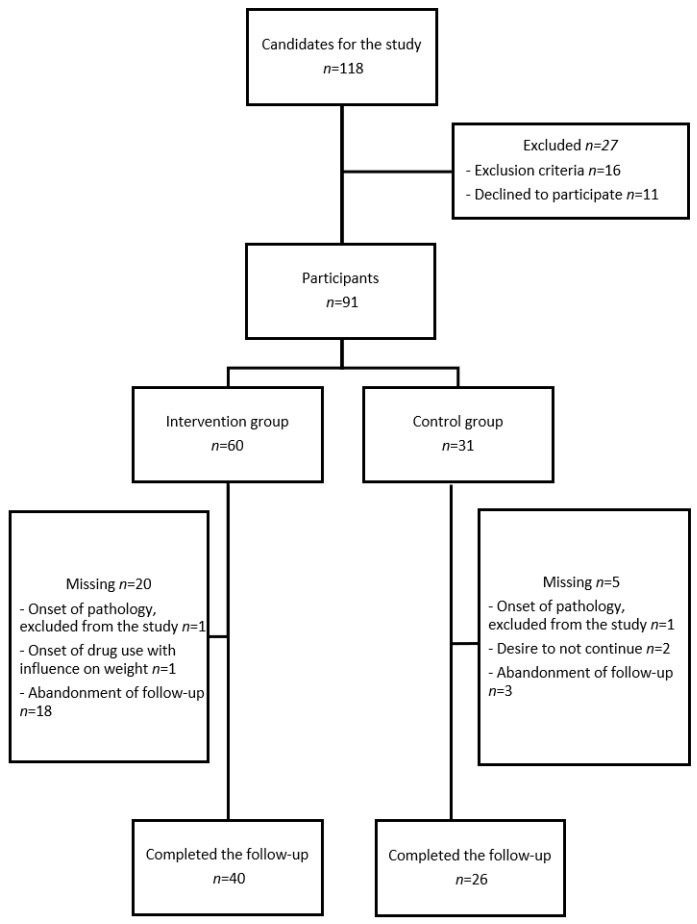
Participant flow chart.

A total of 91 prepubertal children with obesity participated in the study: 60 (65.9%) were in the intervention group and 31 (34.1%) in the control group. During the follow-up, there was a 27.5% dropout rate, with 20 children (33.3%) from the intervention group and 5 children (16.1%) from the control group (Figure 1).

### 3.1. Sample Description

Characteristics of each group are presented in Table 1, and baseline and end-of-follow-up characteristics are shown in Table 2.

### 3.2. Evolution of Patients

#### 3.2.1. Clinical Evolution

In 60.6% of the children who participated in the study, clinically relevant weight loss was obtained with a decrease ≥ 0.5 SDS in the BMI z-score. This was achieved in 60% of the intervention group and in 61.54% of the control group, with no statistically significant differences between the groups. Considering a decrease ≥ 0.25 SDS of the BMI z-score, this was obtained in 63.6% of the total sample (65% of the intervention group and in 61.5% of the control group). The differences between the groups were not statistically significant.

Throughout follow-up, there was a statistically significant decrease (*p* < 0.05) in the median z-IMC, triponderal mass index (TMI) and waist circumference of children in both groups, with no statistically significant differences found between groups.

At the end of follow-up, there were statistically significant differences between the groups, with the median BMI, BMI z-score and IMT being higher in the control group. Likewise, a higher percentage of severe obesity and arterial hypertension was found in the control group, taking into account a homogeneous distribution at baseline.

During the follow-up period, there were no significant changes in blood pressure or in the analytical parameters assessed: HOMA index of insulin resistance (HOMA-IR), interleukin 6 (IL-6), aspartate aminotransferase (AST), alanine aminotransferase (ALT), uric acid, total cholesterol and its fractions and triglycerides.

In the overall sample, between the minors who presented a decrease of ≥ 0.5 SDS in the z-IMC and those who did not, there were no statistically significant differences for the variables sex, age, country of origin, number of cohabitants, family situation (separation of parents or not, single-parent family), socioeconomic level, family history of type 2 diabetes or anthropometry of parents.

#### 3.2.2. Family Grocery Basket Changes During the Follow-Up Period

**Figure 2:**
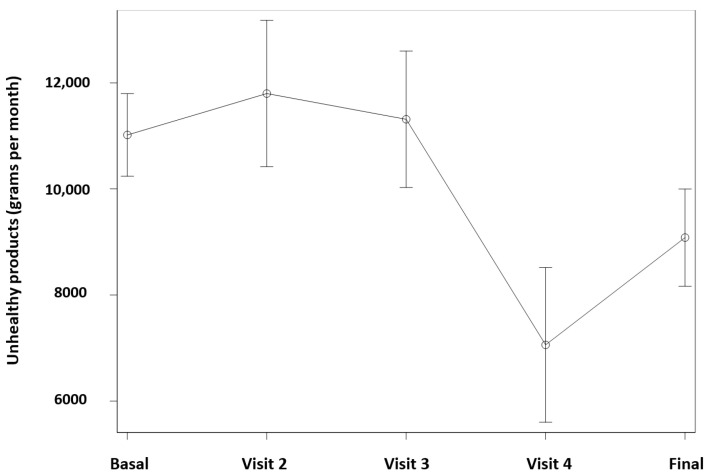
Evolution of the grocery basket expressed in grams of unhealthy product according to the receipts presented at each visit.

The mean consumption of unhealthy products was higher at the initial visits in comparison to the last ones, as shown in Figure 2 (*p* < 0.05).

**Figure 3:**
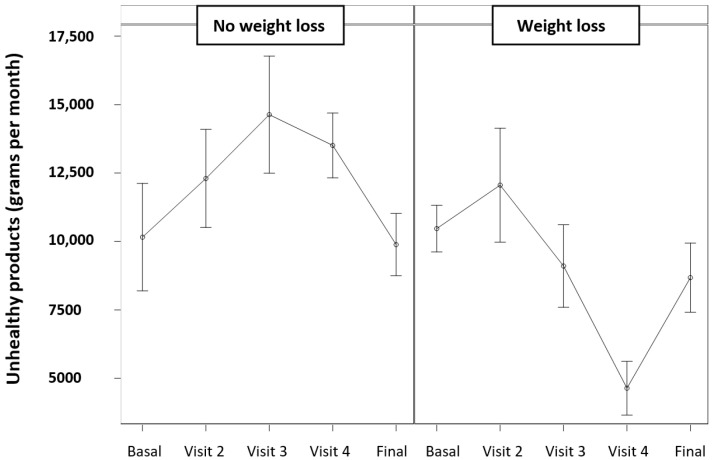
A reduction in the consumption of unhealthy products was observed among children in whom there was a relevant weight loss.

Among children who achieved a relevant weight loss with a decrease of ≥0.5 SDS in the z-IMC, a significant reduction in the consumption of unhealthy products was observed, compared with those who did not achieve such weight loss, as shown in Figure 3.

## 4. Discussion

### 4.1. Relevance of the Study

An intervention focused on the assessment of family grocery basket receipts in the consultation room is an original and novel tool for the family-based treatment of childhood obesity. To our knowledge, this study is the first to analyze the effect of an intervention on the family grocery basket in children with obesity.

There is little scientific literature that refers to the grocery basket in the approach to childhood obesity. Several experts highlight the importance of educating and training parents or caregivers in this area of family management [22,23], and there are general recommendations for healthy shopping, such as going to the supermarket with no hunger sensation or carefully reading the nutritional information labels [22,23]. However, there are no studies that have analyzed the effects of a grocery basket intervention on the evolution of BMI. Recent trials have observed the influence of health strategies on consumers’ decisions, such as proposing healthier alternatives when selecting products for online shopping or clearly displaying the nutritional quality through front-of-pack (FOP) nutrition labels [24,25,26]. However, those studies have not assessed the evolution of BMI or other health parameters of participants.

Clinical practice guidelines advise that the approach to childhood obesity should be based on lifestyle modifications that involve the whole family [14,27,28,29,30,31,32]. Family-based interventions, in addition to being effective in reducing BMI [33,34,35,36,37,38,39], benefit all members of the family household and avoid blaming or stigmatizing children [33].

Our study proposes a practical and simple tool, focusing on family shopping habits in the context of a multi-component family-based treatment of childhood obesity. Grocery receipts, although they do not directly show the caloric intake of children with obesity, are a good indicator of family consumption habits and provide objective and solid data, some of which could be omitted in a survey or in an intake record due to forgetfulness, embarrassment or other reasons. The use of dietary records for the management of childhood obesity, in which all intakes over some days are registered by the child or family, is very widespread in clinical practice. However, given that they require considerable time and effort, it frequently occurs to omit important data or not provide them at all. A recent clinical trial conducted in children and adolescents with obesity explains that measurements of caloric intake were not obtained due to non-compliance in the submission of a four-day intake record [40]. The phenomenon of underreporting in patients with obesity in the pediatric and adult populations is well known [41,42,43].

With the tool we propose, the collection of information is not so tedious, as caregivers only have to keep the receipts and bring them to the consultation. This can be advantageous; moreover, in cases of patients with fragile or vulnerable mental health, it avoids putting them in the position of feeling controlled or guilty about having to record everything they ingest.

We cannot ignore one aspect that can condition the nutritional quality of the family shopping basket, and that is the economic aspect. Although price is a real barrier to maintaining balanced nutrition, especially in vulnerable populations, healthy shopping can be affordable if the right choice of products is made [44]. In Spain, eliminating unhealthy foods from the shopping basket can sometimes be a source of savings, since many of them are not exactly cheap. For example, it is possible to buy high-sugar cereals from a well-known brand for a price eight times higher than healthy oat flakes in the same supermarket. Moreover, in our environment, consuming tap water is cheaper, safer and healthier than any other type of beverage.

### 4.2. Interpretation of Results

The family grocery basket intervention has been shown to be somewhat more effective after one year than usual interventions in the primary care office. The results obtained allow us to validate this intervention as an appropriate and effective tool in the family-based treatment of childhood obesity.

Patients in the intervention group showed a lower percentage of severe obesity and hypertension after one year, based on a homogeneous baseline distribution, showing a greater efficacy of the intervention in the grocery basket ticket compared to the usual ones used in the control group. Clinically relevant weight loss was obtained with a decrease of ≥ 0.5 SDS in the z-IMC in 60.6% of children who participated in the study, achieved in 60% of the group in which the family grocery intervention was performed.

Several studies that have analyzed family-based approaches in childhood obesity corroborate the efficacy of this type of treatment, especially in prepubertal children. For example, a trial conducted in a Spanish population showed the efficacy of a family intervention in improving the BMI and mental health of the participants [38]. Another Spanish study showed a reduction in the hepatic fat mass of children with obesity after 22 weeks of intervention [45]. A team from New Zealand, which conducted a 5-year follow-up study after a 12-month family intervention, observed that the improvement in habits of children with obesity was maintained in the long term, although it was not as successful in terms of BMI evolution, especially in children older than 10 years of age [37]. However, a North American review focused on the Hispanic population concluded that family interventions of a duration of 48 to 144 weeks obtained good results in the evolution of BMI in children with obesity [36]. A Spanish cohort study also obtained positive results, observing a reduction of around 1 SDS in the z-IMC in 62% of children after 2 and 3 years [46]. A recently published North American trial also observed good results in the reduction in BMI in a multi-component family intervention carried out in primary care [8]. A clinical trial conducted in Norway in a hospital setting, analyzing a family-based behavioral treatment in a severely obese child and adolescent population, observed a decrease of ≥ 0.25 SDS in the BMI in 31.5% of the intervention group and in 13% of the control group, which received a routine intervention with visits every three months over one year, as in our study [40]. It is possible that in that study the success rate was lower because it included the adolescent population, in which obesity management is more difficult to manage, and because it was conducted in a hospital and not in primary care [12,47].

All these data, to which our study is added, corroborate the efficacy of the family-based approach as a treatment for childhood obesity in prepubertal children in the primary care office, since this is an ideal environment in which a trusting relationship is established, facilitating adherence to treatment and long-term follow-up when the professionals have the appropriate training and the necessary time to dedicate to this task [48,49].

We have not found an influence of socioeconomic level, family situation (separated parents or not, single-parent family) or the place of origin of the family on the weight evolution of the minors. In the correlation analysis, there were no statistically significant differences in these variables between the children who achieved a significant weight loss and those who did not, despite the abundant literature demonstrating an association between adiposity and social factors, mainly socioeconomic vulnerability [50,51]. We also found no statistically significant association between a family history of diabetes or obesity and the evolution of BMI in children [52]. Larger studies in terms of sample size would make it possible to analyze these relationships more effectively.

About our intervention, it is interesting to note how it had a positive influence on the evolution of shopping habits. Figure 2 shows a statistically significant decrease in the total amount of unhealthy products present in the grocery receipts, with a median of 10,295 g of unhealthy product per month, having decreased in the fourth visit to 46% of the baseline value, reaching 4745 g on the fourth visit (*p* < 0.05). Therefore, our study shows that working with the family grocery basket receipts in the primary care office improves family shopping habits in children with obesity.

In addition, it demonstrated the impact of reducing unhealthy products in the family grocery basket on the evolution of BMI. Among patients who obtained relevant weight loss with a decrease of ≥0.5 SDS in the z-IMC, a progressive reduction in the consumption of unhealthy products was observed, which did not occur among those who did not lose weight (Figure 3). This is in accordance with studies that observed that the degree of compliance with the recommendations correlated with the improvement in z-IMC [52].

Although improvements in parameters such as IL-6 have been described in the literature [35], we found no relevant changes in IL-6 or other analytical parameters during follow-up, probably because most of the children had normal values in the baseline analysis.

### 4.3. Gender Perspective

In our study, although the sex distribution in each group was homogeneous, it is noticeable that only 32% of the sample were female. This is due to the fact that one exclusion criterion for the study was the onset of puberty. As is well known, females usually start pubertal changes at around 9 years of age, and, in addition, these changes can be brought forward in cases of obesity [53]. Males, on the other hand, initiate puberty later, so it was easier to recruit male children.

### 4.4. Limitations

This study has some limitations, such as compliance bias, because there was no masking due to the characteristics of the intervention. This led to several families preferring to participate in the intervention group, hindering random assignment as initially planned. In order to reduce bias, both the evaluation and the intervention of each patient were carried out in both groups by the same professional.

Family grocery receipts are a useful but limited tool. It is possible that families did not come to the consultation with receipts from all purchases, which may have altered data about consumption habits. However, other, more precise, or detailed forms of food recording are also often not adequately completed because of the underreporting phenomenon discussed above. Despite their limitations, grocery basket receipts offer valuable information that provides insight into the eating habits of children and their families.

In our study, 27.5% of participants dropped out of the follow-up, which may have affected the statistical power of the study. It is known that dropout rates in childhood obesity studies are quite high, sometimes reaching over 40% after one year and 60% after two years [52,54].

### 4.5. Future Research

- This study is the first to analyze a family grocery basket intervention for the treatment of childhood obesity. Therefore, further trials analyzing this type of intervention in larger sample sizes of multicentric studies should take into account high percentages of dropouts in order to obtain more powerful results.
- It would be advisable to analyze the use of family grocery basket receipts as a strategy for the prevention of obesity and other pathologies, both in the healthy population and in overweight children.
- Although good results during the first year of follow-up seem to be predictors of a good evolution of BMI during the following years, and the dropout rates in childhood obesity studies are very high, reaching over 60% at 2 years [52], it would be interesting to know the long-term evolution of the children in whom this one-year family intervention has been carried out.

## 5. Conclusions

- The intervention in the family grocery basket through the review of grocery receipts is an easy and effective tool for the treatment of childhood obesity in prepubertal children, improving the shopping habits of families and the evolution of z-BMI SDS.
- The results of this study, with the high percentage of minors who achieved a relevant weight loss, together with the data from other studies, demonstrate the efficacy of family-based interventions in childhood obesity, highlighting the need to invest resources in primary care in order to address this highly prevalent pathology.

## Figures and Tables

**Table 1:** Demographic data in control and intervention groups.

	Control Group N = 31 (34.1%)		Intervention Group N = 60 (65.9%)		*p*
Sex	Girls 35.5%/Boys 64.5%		Girls 30%/Boys 70%		0.59
Age (years old)	7.61 (6.1–9.1) *		8.3 (7.1–9.8) *		0.04
Birth weight (kg)	3.5 (2.9–4) *		3.3 (3–3.7) *		0.48
Length at birth (cm)	50 (47.2–52) *		49.5 (49–51) *		0.56
Place of origin of the family	Spain Latin America Morocco	67.6% 25.9% 6.5%	Spain Latin America Morocco	50% 38.3% 11.7%	0.16
Family situation	Nuclear family Separated parents Single parent	56.7% 40% 3.3%	Nuclear family Separated parents Single parent	76.7% 23.3% 0%	0.07
Number of cohabitants	4 (3–4)		4 (4–5)		0.03
Socioeconomic level	Under Medium High	89.5% 10.5% 0%	Under Medium High	76.3% 18.6% 5.1%	0.27
FH DM2	60%		62.7%		0.62
BMI mother (kg/m)^2^	29.7 (25.1–34) * Normal weight: 25% Overweight: 28.6% Obesity: 46.4%		30.3 (24.7–34.8) * Normal weight: 25.9% Overweight: 22.4% Obesity: 51.7%		0.7
Parent BMI (kg/m)^2^	30.5 (27.3–36.3) * Normal weight: 12% Overweight: 28% Obesity: 60%		28.2 (25.2–31.2) * Normal weight: 24.1% Overweight: 25.9% Obesity: 50%		0.08

Data are shown as percentages (%), mean ± SDS and * median (IQR). FH, family history; cm, centimeters; DM2, type 2 diabetes mellitus; BMI, body mass index; kg, kilograms; m^2^, square meters. Severe obesity was considered in cases of z-IMC > 3.5 SDS.

**Table 2:** Baseline and final characteristics in control and intervention groups.

	Basal			Final		
	Control Group	Intervention Group	*p*	Control Group	Intervention Group	*p*
Weight (kg)	45.8 (37.8–54.6) *	43.7 (37.6–53.8) *	0.65	50.4 (41.5–65) *	47 (40.5–60.2) *	0.61
Size (cm)	132.5 (123–140) *	134.5 (127–142.2) *	0.33	137.6 (129.5–146) *	145 (135.4–150.5) *	0.11
BMI (kg/m)^2^	26.5 (23.6–29.3) *	25 (22.9–27) *	0.06	27.6 (24.8–29.6) *	24.3 (21.4–28) *	0.02
BMI (SDS)	4.65 (3.1–5.6) *	3.31 (2.7–4.5) *	0.01	4.23 (3.1–4.9) *	2.76 (1.7–3.8) *	0.004
TMI (kg/m^3^)	20 (18.2–21.5) *	18 (17.1–20.3) *	0.01	19.2 (18.5–20,6) *	17.6 (15.5–18.9) *	0.001
Waist circumference (cm)	81.7 (75.5–94.5) *	80 (77–88) *	0.24	91 (80.5–99) *	81.8 (76–92) *	0.07
TAS (mmHg)	106 (95–112) *	107 (103–113) *	0.79	113 (103–116) *	109 (107–116) *	0.99
TAD (mmHg)	71.2 ± 8.3	68.1 ± 8.5	0.17	73 ± 9	70 ± 7.9	0.16
Severe obesity	68.4%	45%	0.07	64.3%	32.5%	0.04
HTA	36.8%	20.3%	0.15	50%	13.3%	0.02
Acanthosis nigricans	10.5%	10%	0.94	0%	0%	-
Tanner > 1	0%	0%	-	71.4%	42.5%	0.06
Glucose (mmol/L)	4.7 ± 0.5	4.5 ± 0.4	0.12	4.6 ± 0.5	4.6 ± 0.4	0.9
Insulin (µUI/mL)	18.5 (11.8–28) *	13.5 (10.2–21.3) *	0.22	18.2 (15.7–24) *	12 (9.5–24) *	0.13
HOMA-IR	3.8 (2.7–5.1) *	2.9 (2–4.5) *	0.11	4 (3.1–4.4) *	2.4 (1.9–5) *	0.12
IL-6 (pg/mL)	3.3 (2.1–4.9) *	2.5 (2–4.4) *	0.14	2.4 (2–4.6) *	2.9 (2–4.2) *	0.43
AST (U/L)	24 (21.6–29) *	23.4 (21–27.6) *	0.6	24 (22–25.8) *	25.5 (22.2–29.4) *	0.14
ALT (U/L)	19.8 (15.6–28) *	15.6 (13.2–19.8) *	0.01	20.4 (15–24) *	18.6 (14.5–24.3) *	0.78
Uric acid (mg/dL)	4.4 ± 0.8	4.5 ± 0.8	0.68	4.3 ± 0.9	4.4 ± 1.1	0.67
Cholesterol (mg/dL)	148.3 (133–168.6) *	155.2 (141.9–173.9) *	0.11	142.5 (126.4–151.9) *	161.8 (142.3–176.7) *	0.04
HDL (mg/dL)	46.7 ± 9.26	51.9 ± 10	0.03	47.8 ± 10	50.8 ± 10.7	0.33
LDL (mg/dL)	87.8 (73–104.4) *	94.9 (77.1–107.8) *	0.39	77.2 (68.4–101.5) *	95.7 (80.6–108) *	0.11
TG (mg/dL)	77.9 (51.6–98.9) *	75.3 (59.5–111.1) *	0.67	75.8 (56–92.8) *	67.8 (60.4–94.5) *	0.94

Data are shown as percentages (%), mean ± SDS and * median (IQR). ALT, alanine aminotransferase; AST, aspartate aminotransferase; HOMA-IR, HOMA index of insulin resistance; HT, hypertension; BMI, body mass index; BMI (SDS), BMI in standard deviations; TMI, triponderal mass index.

## Data Availability

Data are available upon request.
